# Electronic and structural properties of the natural dyes curcumin, bixin and indigo

**DOI:** 10.1039/d0ra08474c

**Published:** 2021-04-19

**Authors:** Leander Michels, Annika Richter, Rajesh K. Chellappan, Håkon I. Røst, Alenka Behsen, Kristin H. Wells, Luciano Leal, Vilany Santana, Rosana Blawid, Geraldo J. da Silva, Simon P. Cooil, Justin W. Wells, Stefan Blawid

**Affiliations:** Department of Physics, Norwegian University of Science and Technology (NTNU) 7491 Trondheim Norway; Center for Quantum Spintronics, Department of Physics, Norwegian University of Science and Technology (NTNU) 7491 Trondheim Norway quantum.wells@gmail.com; Department of Clinical and Molecular Medicine, Norwegian University of Science and Technology (NTNU) 7491 Trondheim Norway; Department of Materials Science and Engineering, Norwegian University of Science and Technology (NTNU) 7491 Trondheim Norway; Department of Electrical Engineering, University of Brasília 70910-900 Brasília Brazil; Department of Agronomy, Federal Rural University of Pernambuco 52171-900 Recife Brazil; Institute of Physics, University of Brasília 70910-900 Brasília Brazil; Department of Physics, University of Oslo Sem Slands Vei 24 7491 Oslo Norway; Center for Informatics, Federal University of Pernambuco 50740-560 Recife Brazil

## Abstract

An optical, electronic and structural characterisation of three natural dyes potentially interesting for application in organic solar cells, curcumin (C_21_H_20_O_6_), bixin (C_25_H_30_O_4_) and indigo (C_16_H_10_N_2_O_2_), was performed. X-Ray Diffraction (XRD) measurements, showed that curcumin has a higher degree of crystallinity compared to bixin and indigo. The results from the Pawley unit cell refinements for all dyes are reported. Optical absorption spectra measured by UV-Visible Spectroscopy (UV-Vis) on thermally evaporated films revealed that bixin undergoes chemical degradation upon evaporation, while curcumin and indigo appear to remain unaffected by this process. Combined Ultraviolet Photoemission Spectroscopy (UPS) and Inverse Photoemission Spectroscopy (IPES) spectra measured on the dyes revealed that all of them are hole-conducting materials and allowed for the determination of their electronic bandgaps, and Fermi level position within the gap. UV Photo-Emission Electron Microscopy (PEEM) revealed the workfunction of the dye materials and indicated that indigo has a negative electron affinity. PEEM was also used to study degradation by UV irradiation and showed that they are quite robust to UV exposure.

## Abstract 2

An optical, electronic and structural characterisation of three natural dyes potentially interesting for application in organic solar cells, curcumin (C_21_H_20_O_6_), bixin (C_25_H_30_O_4_) and indigo (C_16_H_10_N_2_O_2_), was performed.

## Graphical abstract

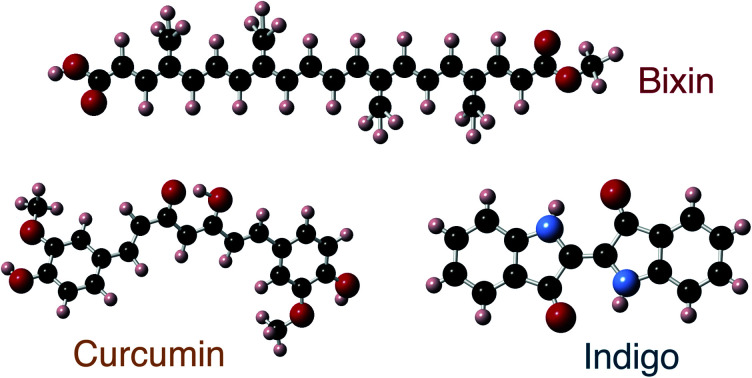

## Introduction

Molecular dyes have become an essential part of dye sensitized solar cells (DSCs), and during the last two decades, significant improvements in efficiency and lifetime have been demonstrated. A significant proportion of these dyes are based on natural organic materials – for example derived from the coloured pigments found in fruit, flowers, and other plant material.^1–13^

Whilst solar cells based on synthesised polymers are currently marking the standard in higher performance organic photovoltaics (OPV),^14–16^ there is a lot of potential in uncovering the suitability of natural materials. Using natural materials in organic photovoltaics, as opposed to synthetic polymers, is appealing considering that the processing of the latter often requires hazardous halogenated solvents.^17^ On top of that, the sustainable harvesting of useful materials from nature could prove to be cost-effective, accessible in developing regions and free from environmental concerns. Some industries have already established this type of sustainable supply chain processes in the Amazon region.^18^

**Fig. 1:**
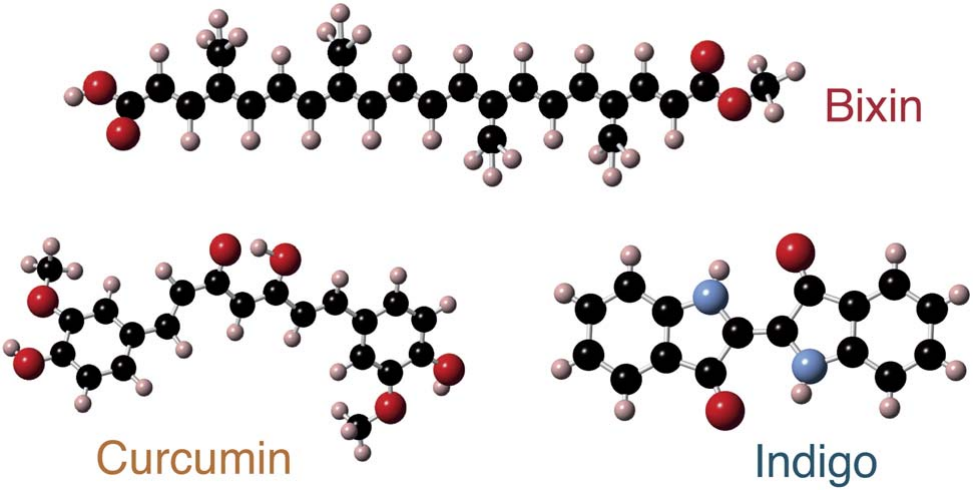
Schematic depiction of three dye molecules: bixin, curcumin and indigo. The bixin molecule is drawn entirely as a schematic, but the curcumin and indigo molecules are the calculated (relaxed) structures; see ‘calculations’ section for details. Other tautomeric forms may also be possible. The molecules contain carbon (black spheres), hydrogen (peach), oxygen (red) and indigo additionally contains nitrogen (blue).

In this work, natural dyes, which due to their strong absorption in the visible spectrum have high potential for application in OPV, were investigated using optical, chemical, electronic and structural characterization methods. Specifically, we investigated three naturally occurring dyes; curcumin (C_21_H_20_O_6_), bixin (C_25_H_30_O_4_) and indigo (C_16_H_10_N_2_O_2_). The structure of the three dye molecules are depicted in Fig. 1. Curcumin is a pigment of turmeric and has been used as a spice and home-remedy against different ailments, most prominently in India, for centuries.^19,20^ Bixin is the main carotenoid found in the seeds of the annatto tree native to South America and is extensively used as a food colourant,^21,22^ while indigo is the common dye used in the production of denim cloth for blue jeans.^23^ These materials have low toxicity (both tumeric and bixin are approved for consumption and labelled as E100 and E160b, respectively, in the European Union), and low price (tumeric and indigo currently have bulk prices around 3–5 US$ per kg, and bixin is approximately an order of magnitude more expensive).

## Experimental

All dyes used in this work were purchased from Sigma-Aldrich in powder form: Indigo (product number 229296), bixin (05989) and curcumin (both 78246 with ≥99.5% purity and curcuma longa powder C1386 with ≥60% purity were used).

Throughout this work, two different sample preparation methods have been used: (1) samples were prepared by thermal evaporation in vacuum using a custom built thermal evaporator, calibrated with an integrated thermocouple to monitor the temperature and (2) samples where prepared by drop-casting dye material dissolved in a solvent (ethanol or chloroform) onto a substrate and allowing the solvent to evaporate. All experimental methods were used on all samples, to allow any dependence on the preparation methods to be observed.

X-ray diffraction was performed at the XRD2 beamline at the Brazilian Synchrotron Light Laboratory (LNLS). The diffraction patterns were collected at a beam energy of 8 keV (*λ* = 1.54978 Å). The dyes were loaded as-supplied into a cavity in a Cu sample holder sealed on one side by 1.5 μm thin Al foil, which was demonstrated to have a high transparency at this photon energy. The scattered X-ray intensities were collected as a function of scattering angle 2*θ*.

UV-visible spectroscopy was performed in order to measure the spectral absorbance. For this experiment dye samples were prepared on 1 mm thick quartz glass holders, because quartz has very low absorbance in the wavelength range of interest (≈300–800 nm).

For studying the chemistry and electronic structure of the dyes, Photoemission Spectroscopy (PES) measurements were carried out in an ultra-high vacuum chamber. The samples were prepared by thermal evaporation on a non-reactive and clean (carbon-free) MoS_2_ substrate, which was cleaved immediately before introducing it into the vacuum chamber. For studying the valence band states, Ultraviolet Photoemission Spectroscopy (UPS) was performed with a UV discharge lamp using the He-Iα emission line at 21.22 eV. For X-ray Photoelectron Spectroscopy (XPS) core-level acquisitions, an unmonochromated Mg-Kα X-ray source (*hν* = 1254 eV) was used. Imaging and UV degradation testing was performed using a NanoESCA PEEM with illumination from a high intensity unmonochromated Hg discharge lamp.

Conduction band states were studied by Inverse Photo-emission Spectroscopy (IPES) in isochromat mode with an instrument built by PSP Vacuum Technology. This instrument uses a variable energy electron gun based on a low temperature BaO secondary electron emitter, and a modified channeltron detector which detects photons of fixed energy (≈9.6 eV).

During XRD, XPS, PEEM, UPS and IPES acquisitions, care was taken to look for (and avoid) conditions under which beam damage of the materials could be occurring.

## Results and discussion

**Fig. 2:**
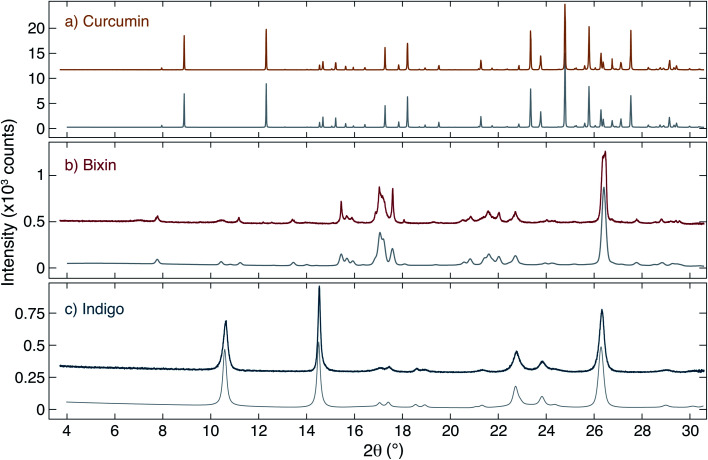
Measured and simulated XRD signals. (a) Curcumin, (b) bixin and (c) indigo: in each case the upper (coloured) trace shows the data collected at the XRD2 beamline of the LNLS, and the lower trace (grey) shows simulated patterns obtained based on Pawley unit cell refinement. The structural parameters used are described in Table 1.

The structure of the dyes was studied using X-ray diffraction. The measured diffraction patterns of curcumin, bixin and indigo together with simulated patterns obtained from a Pawley unit cell refinement^24,25^ are shown in Fig. 2. Curcumin is found to show a significantly higher crystallinity (*i.e.* larger crystallites) than the other two dyes, as evidenced by the Bragg peak intensities and widths.

As a starting point for the Pawley refinement, the crystal structures found by Reid *et al.*^26^ (curcumin), Kelly *et al.*^27^ (bixin) and Süsse, Wolf *et al.*^28,29^ (indigo) were used. In the case of indigo, both polymorphs indigo A and B (whose unit cells differ only slightly except for the parameter *β*) are expected to be present in the sample with the vast majority being indigo B. The refined unit cell parameters for all crystals are listed in Table 1. In all cases, the structure was found to be in reasonably good agreement with literature values and only a small refinement of the structural parameters from ref. 26–31 was needed. The geometric parameters used to refine the curcumin crystal structure were fixed in the refinement of indigo and bixin samples.

**Table 1:** Refined lattice parameters of curcumin, bixin and indigo A and B obtained from Pawley unit cell refinement of measured XRD patterns and crystallite size analysis from Pawley fits. Indigo A phase occurs only in small amounts, and this makes the analysis have a higher uncertainty. Bixin has a very weak signal so the uncertainty is also relatively large. Note: uncertainties may be underestimated. Asterisk (*) indicates that the value is fixed to the literature value^29^

	Curcumin	Bixin	Indigo A	Indigo B
Space group	*P*2/*n*	*P*1̄	*P*2_1_/*c*	*P*2_1_/*c*
*a* [Å]	12.7434(1)	11.479(2)	9.387(5)	10.939(2)
*b* [Å]	7.2207(2)	12.000(2)	5.682(3)	5.853(1)
*c* [Å]	20.056(1)	8.916(1)	11.898(7)	12.300(1)
*α* [°]	90	104.15(2)	90	90
*β* [°]	94.991(2)	93.37(1)	117*	130.20(2)
*γ* [°]	90	91.27(1)	90	90
Size [nm]	710 ± 20	84 ± 5	3.2 ± 0.2	37 ± 3

The solid-state structure of curcumin in the monoclinic space group *P*2/*n* was first reported in 1982 by Tønnesen,^32^ and later by several others.^26,33–35^ Additional polymorphs of curcumin have also been reported, *i.e.* keto–enol tautomers, low temperature phases and possibly other polymorphs.^34–36^ On the other hand, our finding of the common *P*2/*n* phase is uncontroversial as the main form in simple dye extractions such as ours.

The available literature regarding the structure of bixin and indigo is less plentiful and generally uncontroversial. Our refined structural parameters for indigo differ by only a few percent from that of Süsse, Wolf *et al.*,^28,29^ and our XRD results are visually similar to ref. 37. In the case of bixin, Pinzón-García *et al.* studied bixin compounds for biomedical applications found that their material to be a semi-crystalline solid.^38^ This is significantly different from the results reported here, which show a higher degree of crystallinity. A direct comparison between our XRD data and the data in ref. 38 reveals that the same peaks are present in both studies, however in ref. 38 the peaks are much weaker, broader and fit on top of a ‘humped’ background. This background intensity is large compared to the peak intensities and is maximal at 2*θ* ≈ 20°. In our measurements, the background intensity is low and unstructured, and the peaks are significantly sharper. We interpret this to mean that we essentially see a similar crystal structure but that our crystallite size is significantly larger; *i.e.* the data is ref. 38 are indicative of very small crystallites and hence their sample is described as a ‘semi-crystalline solid’. We suggest that this is probably a consequence of differences in the sample preparation which can cause the crystallisation of organic materials to be quite distinct.

**Fig. 3:**
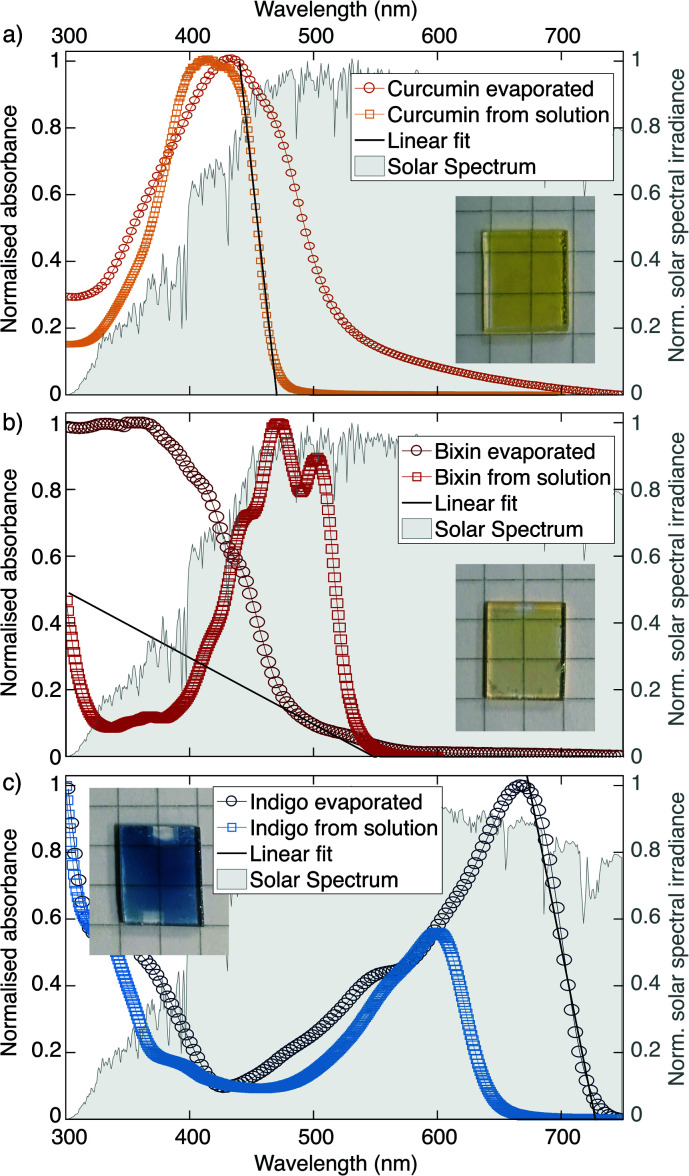
UV-visible Absorption spectra. (a) Curcumin, (b) bixin and (c) indigo measured on evaporated films and in films prepared from solutions of the dyes in chloroform. Photographs of the evaporated samples are also shown.

Optical absorption spectra measured by UV-Visible Spectroscopy are shown in Fig. 3 for the three dyes used in this study. For each dye, measurements were performed both on a film which had been thermally evaporated on a quartz glass substrate as well as from a drop-cast solution of the dye in chloroform. The measured spectral absorbance was normalised in order to better compare the samples independent of film thickness. The graphs are underlaid with the AM 1.5 spectrum of solar irradiance (data from ref. 39). For visualisation purposes photographs of the evaporated films are also shown as insets.

In the case of curcumin and indigo, the spectra measured on the evaporated films are shifted to higher wavelengths compared to the measurements of the solutions, which can be explained by supramolecular polymerisation, as first described by Scheibe *et al.*^40^ In the case of bixin, however, the spectrum of the evaporated film is shifted to shorter wavelengths, which is in line with the visibly yellow colour of the sample (as opposed to the red powder from which it is made). This suggests that bixin undergoes chemical degradation under the influence of the high temperatures involved during evaporation. We therefore infer that bixin is damaged by evaporation, but curcumin and indigo appear not to be.

From the absorption spectra presented in Fig. 3 estimations for the optical bandgap of the dyes were derived (see Table 2). The maximum wavelength at which the molecules absorb was determined by placing a linear fit on the absorption edge and finding the intersection with the x-axis. The prediction of the absorption edge could be slightly improved by applying the so-called Tauc relation.^41^ The strong shift of the absorption edge of the evaporated bixin film towards shorter wavelengths corresponds to an increased bandgap as compared to the drop cast sample. Since a larger bandgap can generally be associated with a shorter conjugation length this could be an indicator that the evaporation causes the long conjugated chain of the molecule to break apart.

**Table 2:** Overview of the workfunction (obtained from the secondary cutoff observed with UPS/PEEM), optical bandgaps (obtained from UV-Vis absorption spectra) and electronic bandgaps (measured by photoemission spectroscopies UPS and IPES), together with the DFT calculated optical and HOMO–LUMO gaps. Note: the curcumin calculations are performed on ‘trans’ molecules (*i.e.* differing from the model if Fig. 5 by the placement of the central H atom), however this does not play an important role in the *E*_g_ calculation. Note also that the UPS-IPES bandgap uncertainty is an optimistic estimate

	Empirical				Calculated	
	Workfunction [eV]	*E*_g_ optical [eV]		*E*_g_ electronic [eV]	*E*_g_ optical [eV] (single molecule)	*E*_g_ [eV] (HOMO–LUMO)
		Evaporated	In solution	UPS/IPES		
Curcumin	3.2 ± 0.1	2.33 ± 0.02	2.64 ± 0.01	2.3 ± 0.1	3.08	3.60
Bixin	3.3 ± 0.1	2.5 ± 0.1	2.33 ± 0.02	2.8 ± 0.1	2.62	2.22
Indigo	1.7 ± 0.1	1.71 ± 0.01	1.92 ± 0.01	1.9 ± 0.1	1.81	2.05

**Fig. 4:**
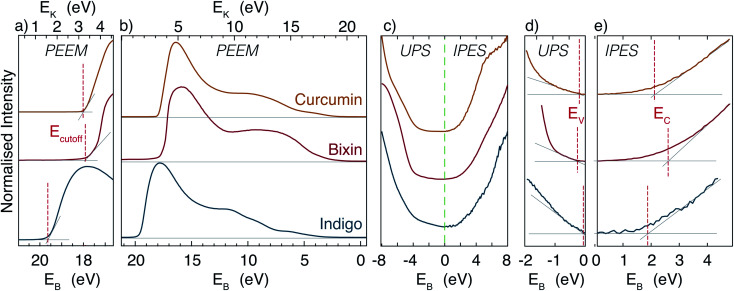
Combined PEEM, UPS and IPES spectra of curcumin, bixin and indigo. (a) Detail of the low energy secondary cutoff, collected using PEEM (with the extracted value of *E*_cutoff_ indicated) and (b) the full bandwidth from *E*_cutoff_ to the Fermi level. (c) An overview of the states near to the bandgap for the three dyes. A zoom-in on the detail of (d) the valence band maxima and (e) the conduction band minima from the three dyes (coloured traces), together with the linear fits/extrapolations (fine grey lines) applied to extract the valence and conduction band edges. The vertical dashed red lines indicate the extracted values. All data is from samples prepared by in vacuum evaporation, except the UPS measurement of bixin: sample degradation by the UV source precluded this possibility and so this measurement is performed on a sample prepared from solution. Despite this precaution, the unusually large value of the conduction band minimum is thought to be indicative of sample degradation.

The electronic bandgap has also been measured using UPS and IPES. The UPS and IPES spectra for each dye are displayed together in Fig. 4. The graphs include linear fits applied to determine the valence and conduction band edges. The tail of the IPES data is a result of the gradual onset of photoionisation in the sodium chloride coated photocathode in the detector, and is also seen when measuring a polycrystalline Ag reference sample. It is therefore not considered to be primary signal, and is ignored. As can be seen from the figure, there is a moderate uncertainty involved in the extraction of the band extrema, due to the fact that the signal reduces in a nonlinear manner. This is an indication that the composition of the sample is not uniform; *i.e.* that it may have non uniform doping (from impurities such as water), *etc.*

In all three dyes the valence band edge is found to be much closer to the Fermi level than the conduction band edge. This means that the materials are p-type in character, *i.e.* that they are hole-conducting materials, as is the case for most organic semiconductors.^42^

The electronic bandgaps calculated as the difference between the valence and conduction band edges are listed in Table 2. The large bandgap observed in bixin stems mostly from the high conduction band edge, which is in line with the likely damaging of the molecule in the evaporated film on which the IPES measurement was performed. Additional uncertainty in the spectra of bixin is added by the fact that the films were very thin. As a result of this, contributions from organic contaminants cannot be ruled out, and that the UPS-IPES bandgap extraction for bixin is not completely reliable. However, for curcumin and indigo, the UPS-IPES bandgap analysis is reliable, and agrees well with the UV-vis measurement, indicating that the electronic and optical bandgaps are similar. Furthermore, the UPS-IPES also reveals the Fermi level position, and hence shows the relatively strong natural p-type nature of these dyes. Finally, the difference between the electronic bandgap and the optical gap is in general caused by the exciton binding energy. From the UV-Vis of evaporated films and the UPS/IPES measurements we would estimate exciton binding energies of ≈0 eV and 0.2 eV for curcumin and indigo, respectively.

The energy of the low energy secondary cutoff *E*_cutoff_ has also been extracted from UPS measurements (carried out using a biased sample in a PEEM instrument), and is shown in Fig. 4(a), together with the full bandwidth of the UPS signal in Fig. 4(b). From these measurements, it is straightforward to estimate the sample workfunction (*i.e. E*_cutoff_ relative to the Fermi energy). These values are shown in Table 2. For all dyes, the workfunction is surprising low, and in the case of indigo, the value we find is exceptionally low. In fact, for indigo, the workfunction of ≈1.7 eV is less than both the bandgap and the energy of the CBM, thus indicating that the electron affinity is negative. A negative electron affinity could potentially be useful for discouraging the process of electron–hole recombination, which is detrimental to PV efficiency.

**Fig. 5:**
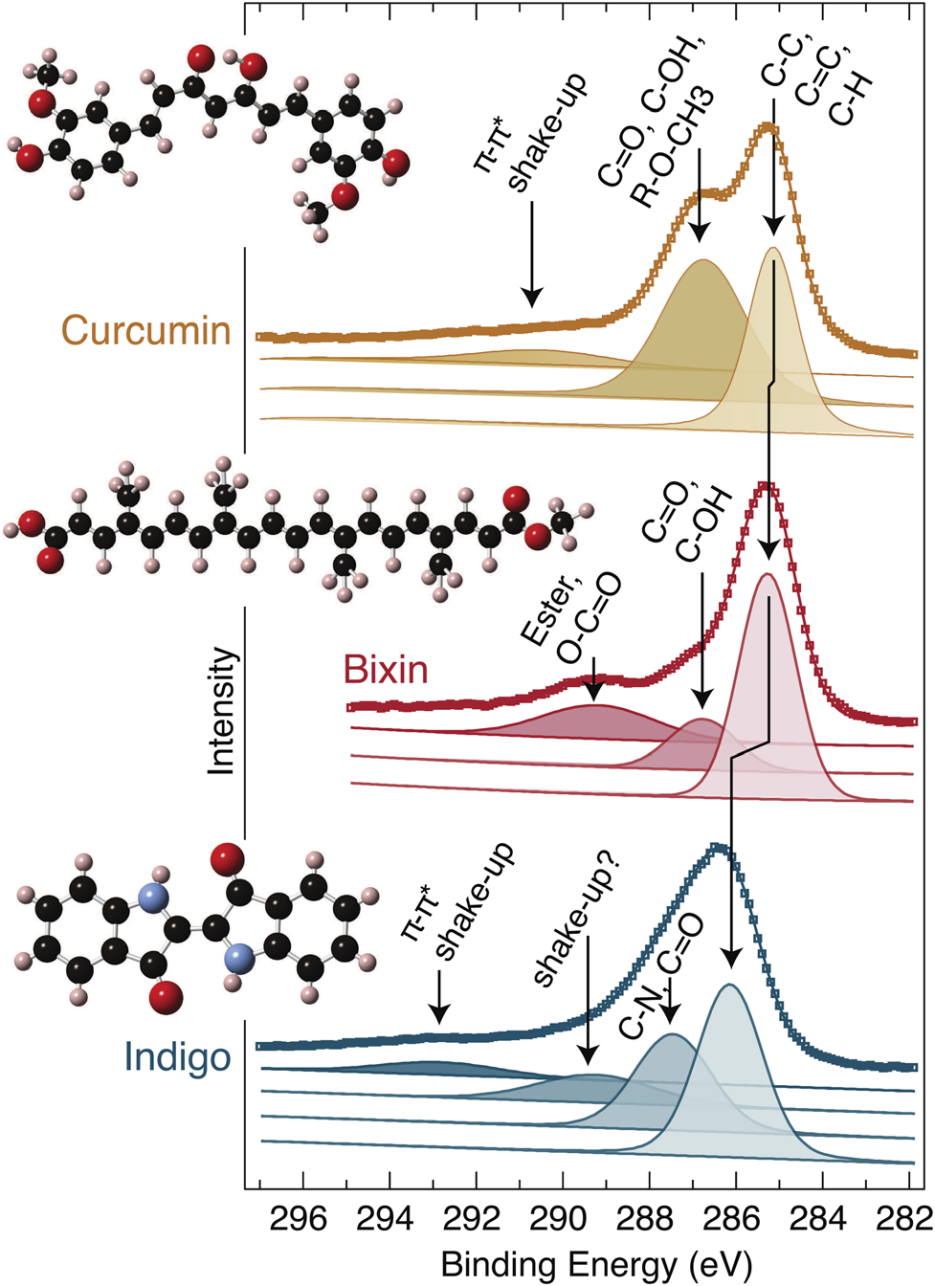
XPS measurements. The C 1s spectra of curcumin, bixin and indigo. The curcumin and indigo samples were prepared by in-vacuum thermal evaporation, but the bixin sample was necessary prepared from solution. The raw data in presented using coloured squares together with fitted envelopes (solid lines) and the separated individual components from which the fits are comprised. Each component is labelled to indicate its chemical origin. The structure of the molecules are shown as insets to the figure. Note: for curcumin^26^ and indigo^50^ the calculated structure is used, but for bixin the inset is a schematic. All data are collected with a Mg-Kα X-ray source.

XPS core level measurements have also been performed, and are plotted in Fig. 5. Widescans were performed to check for contamination, but only the expected components (*i.e.* C, N and O) were seen. The C 1s peak is of particular interest, and hence these sample were prepared on a freshly cleaved MoS_2_ substrate; on which it is straightforward to prepare a new surface which is free of carbon.^43^ Furthermore, being a van der Waals layered material, the surface contains no dangling bonds, and has been seen to be unreactive towards organic material.^43^ The C 1s components have a rich structure due to the large number of inequivalent carbon atoms contained by each molecule. It is not feasible to deconvolute all of the individual carbon environments due to their small separation and broadness, but it is possible to identify groups of similar atomic environments. In Fig. 5, both the raw data (square markers) and fitted curves (continuous lines) are plotted, and the individual components which contribute to the overall fit are shown below. The tallest peak in each case is the lowest binding energy component; due to the C–C, C=C and C–H bonding which are prevalent in all three molecules. The neighbouring peak at higher binding energy (≈287–288 eV) is caused by carbon with an alcohol, aldehyde or ketone group attached, as well as the ether group (in the case of curcumin) and nitrogen neighbour (in the case of indigo). Additionally, curcumin and indigo exhibit a π–π* ‘shake-up’ at high binding energy (EB ≈ 291 and 293 eV, respectively), which is due to excitations between the filled and empty conjugated π states of the aromatic rings. Bixin also has a small higher binding energy peak (EB ≈ 290 eV) which is due to the ester and carboxylic carbon atoms. Finally, indigo exhibits a small and broad peak (EB ≈ 289 eV) which we are unable to definitively explain, but suggest that it may also be due to a shake-up process.

The bixin C 1s components presented here are consistent with those published by Felicissimo *et al.*,^44^ however there is a systematic shift in the binding energies. This could be due to differences in doping, instrument calibration, contamination or sample preparation.

Our assignment of the shake-up features^45^ and carbon components has been based on published literature of a range of carbon containing materials, *i.e.* ref. 43 and ^46–49^. Our analysis shows that, except for a small component in the indigo C 1s (which may be due to an additional shake-up process), all of the constituent components can be identified and matched with the expected chemical environments. *i.e.* this offers additional confirmation that the dyes have not been degraded during the preparation and XPS measurements.

## Calculations

In addition to measuring the optical and electronic gap, we also computed the optical gap of the three dyes for a single molecule in vacuum. To achieve this, first the molecular geometry was optimised *via* density functional theory (DFT) employing a B3LYP functional with a 6-311+G(d,p) basis set. Although DFT is a ground state theory, we report also as reference the energy differences found between the highest occupied molecular orbital (HOMO) and the lowest unoccupied molecular orbital (LUMO). However, the HOMO–LUMO gap does not serve as an estimate of the fundamental gap, which is substantially underestimated by DFT.^51^ Freezing the optimised geometry, the charge density response to a weak time-periodic external potential is computed *via* time dependent (TD) DFT. Fifty entries were considered in the coupling matrix between occupied and unoccupied states and the optical gap determined as the lowest dipole-allowed eigenvalue. For the TD-DFT computations the hybrid exchange–correlation functional CAM-B3LYP is used. The results are summarised in Table 2.

In case of curcumin, the computed single-molecule optical gap overestimates the values extracted from UV-vis both for evaporated samples (by ≈30%) as well as for the dyes in solution (by ≈17%). There are several potential reasons for this; for example, curcumin exists in different tautomeric forms and previous studies^52^ have reported that the calculated gap is significantly different for the enol and keto forms (*i.e.* with and without a central hydrogen in the bridge site between the two O groups). It is also important to point out that the calculated values depend on the basis set used: we use B3LYP/6-31+G(d,p) whereas in the study by Abduljalil *et al.* a number of different basis sets are used.^52^ On the other hand, Abduljalil *et al.* show results using 6-31+G(d,p) (which should be closely comparable with our calculations) and report HOMO–LUMO gap values of 3.60 eV (keto) and 3.25 eV (enol). In other words, whilst it is questionable whether the enol or keto form is the most appropriate to use, DFT nonetheless yields a value of *E*_g_ which is significantly higher than our experimental findings. We believe that this discrepancy may be an indication that single-molecule calculations do not adequately describe the experimental case. This is fully consistent with the XRD analysis which shows that curcumin forms large (*i.e.* 300 nm) crystallites. In other words, the long range order present in the experimental films cause a significant deviation from the single-molecule case.

In the case of indigo the computed single-molecule optical gap agrees surprisingly well with the empirical value extracted from UV-vis for the evaporated sample (only 6% overestimation). This points to only weakly interacting indigo molecules in the film. In addition to our own findings, we point out that in the work of Irimia-Vladu *et al.* a variety of methods are used and the optical and electronic gaps are reported.^37^ Their reported values (all of which are around 1.7 eV) are at the lower end of our range, but in general the agreement between our work and theirs is very satisfactory. From the comparison of the optical gap extracted from UV-vis for evaporated samples and the bandgap extracted from UPS/IPES, an estimation of the exciton binding energy is possible. The difference gives ≈0 eV and 0.2 eV for curcumin and indigo, respectively. The very weakly coupled electron–hole pair in curcumin is interesting since it should facilitate charge separation in photovoltaic devices. If the finding is related to the good crystallinity of curcumin evidenced throughout the present work remains to be investigated.

For both indigo and curcumin, reliable measurements could be performed on evaporated samples. In the case of bixin, this does not seem to be possible because the thermal evaporation appears to significantly degrade the molecule, evidenced for example by a change in colour. Furthermore, whilst it appeared that XPS and UPS measurements could be performed without causing damage, degradation during the IPES measurement is likely. Thus, the most reliable empirical value for bixin is the optical gap of 2.33 eV measured in solution. If we assume a similar redshift as observed for the other two dyes, an optical gap in the order of 2.1 eV may be inferred for a hypothetical non-degraded bixin film. Thus, in comparison with the computed single-molecule optical gap of 2.62 eV, intermolecular interactions appear to be significant in bixin as in the case of curcumin.

## Ageing due to UV exposure and heat

In order to evaluate the robustness of natural dyes in PV applications, we have also tested their response to UV exposure and heat. In addition to the He discharge lamp used in our PEEM instrument for the valence band and workfunction measurements, the instrument is also equipped with a high intensity Hg discharge lamp (photon energy ≈5 eV). We estimate the photon flux in the visible and UV range to be ≈×10^4^ higher than solar flux: in other words, 1 h of exposure corresponds to ≈2 years of 12 h per day exposure to sunlight.

We conducted an experiment in which we first collected the full valence band and secondary cutoff using He illumination (*i.e.*Fig. 3(a) and (b)). We then left the sample exposed to high intensity illumination from the Hg lamp for up to 3 h (*i.e.* equivalent to ≈6 years of operation in sunlight), whilst occasionally remeasuring the valence band and secondary cutoff measurements to look for signs of damage. We have reported a similar approach for accelerating UV induced changes previously.^43,53^

Following 2–3 h of Hg illumination, no dramatic changes are observed in the valence band structure. The VB intensity of bixin is seen to reduce by ≈20%, relative to the pristine sample. For indigo, the intensity reduction is less notable at ≈10%, and for curcumin no reduction is seen at all. All three dyes keep the same VB shape indicating that no dramatic change in bonding has occurred. All 3 dyes show a change of workfunction: curcumin and bixin show an increase of ≈0.5 eV whereas indigo shows a decrease of about the same magnitude. In short, after 2–3 h of exposure to an intense Hg source (simulating ≈6 years of operation in sunlight), some signs of degradation are seen in all three dyes. In all three cases, the degradation is not severe, and it is least noticeable in curcumin.

In addition to UV exposure, we have also tested the robustness to heating: Bixin appears to be damaged at quite low temperatures, but survives temperatures of at least ≈40 °C. Both curcumin and indigo can be heated to their evaporation temperature (≈170 °C without damage, as evidenced by the fact that the evaporated samples are extremely similar to samples prepared from solution. In other words, the dyes can generally survive temperatures sufficient for most PV applications, but bixin is least robust in this regard.

## Conclusion

We have performed a comprehensive study of three natural dyes: curcumin, bixin and indigo. The material used here has been purified to a high standard, and the purpose of this work is to reveal the intrinsic structural, chemical and electronic properties of the selected dyes, and to consider their potential as components in an organic photovoltaic structure. In larger quantities, these materials are cheap (currently the market price for tumeric is 3 USD per kg, and indigo 5 USD per kg), they are also non-toxic (curcumin E100 and bixin E160b are both safe to consume), abundant and present no significant environmental concerns. In many respects, they are ideal candidates for ‘socially responsible’ photovoltaics.

Our work has utilised a wide range of experimental methods (X-ray diffraction, UV-visible absorption spectroscopy, X-ray photoelectron spectroscopy, UV photoemission spectroscopy, photoemission electron microscopy and inverse photoemission spectroscopy) combined with TD-DFT calculations. We have studied both thermally evaporated thin films, and samples prepared from solution. In line with the theme of low-budget and accessible materials, these preparation methods are also low budget and readably scalable. All three dyes were found to have bandgaps in a suitable range for PV applications (*i.e.* ≈1.7 to 2.8 eV) and low workfuctions. Indigo was found to have an unusually low workfunction, indicating a negative electron affinity.

We report that all three materials can be satisfactorily prepared using low budget methods. However, whilst curcumin and indigo can easily be thermally evaporated without any sign of degradation, bixin needs to be handled more carefully. The thermally evaporated films of curcumin and indigo appear to be uniform, flat, continuous, and generally high quality and ‘well behaved’. Not only is bixin degraded by thermal evaporation, but our measurement methods (especially IPES) may also cause degradation. Whilst this can be circumvented for the case of this study, it raises the question of its stability (and therefore suitability) in a real photovoltaic. Presumably some form of encapsulation would be used,^54^ which may act to protect the dye molecules, but the fact that bixin is relatively easy to damage serves as a warning sign. Additional studies of its ageing under UV exposure would be straightforward to carry out^53^ and could assist in concluding whether it is robust enough for applications involving extended UV exposure. In this regard, curcumin and indigo are of least concern since no sign of degradation was seen, even after significant exposure to high intensity UV.

The efficiency (and feasibility) of using natural dyes in photovoltaics has attracted significant attention. Whilst the reported efficiency for cells utilising these particular dyes is low (bixin on a TiO_2_ substrate has shown 0.53% (ref. 10) and curcumin, mixed with red cabbage, has been reported to return a 0.6% efficiency^55^), the easy accessibility of the materials and preparation methods, coupled with the low toxicity, low cost and low environmental impact, makes such photovoltaics especially appealing in developing and ‘off-grid’ markets, and for low power applications.

Finally, our conclusion is that the robustness of curcumin and indigo, together with their ‘close to optimal’ bandgaps and doping make them the best candidates that we have studied so far in the search for natural materials for photovoltaic applications. Moreover, curcumin revealed an exceptionally low exciton binding energy. Whilst it may be possible to find even better candidates, our findings reassure us that low cost, socially and environmentally responsible photovoltaic for low power applications are certainly feasible.

## Conflicts of interest

There are no conflicts to declare.

## Supplementary Material
